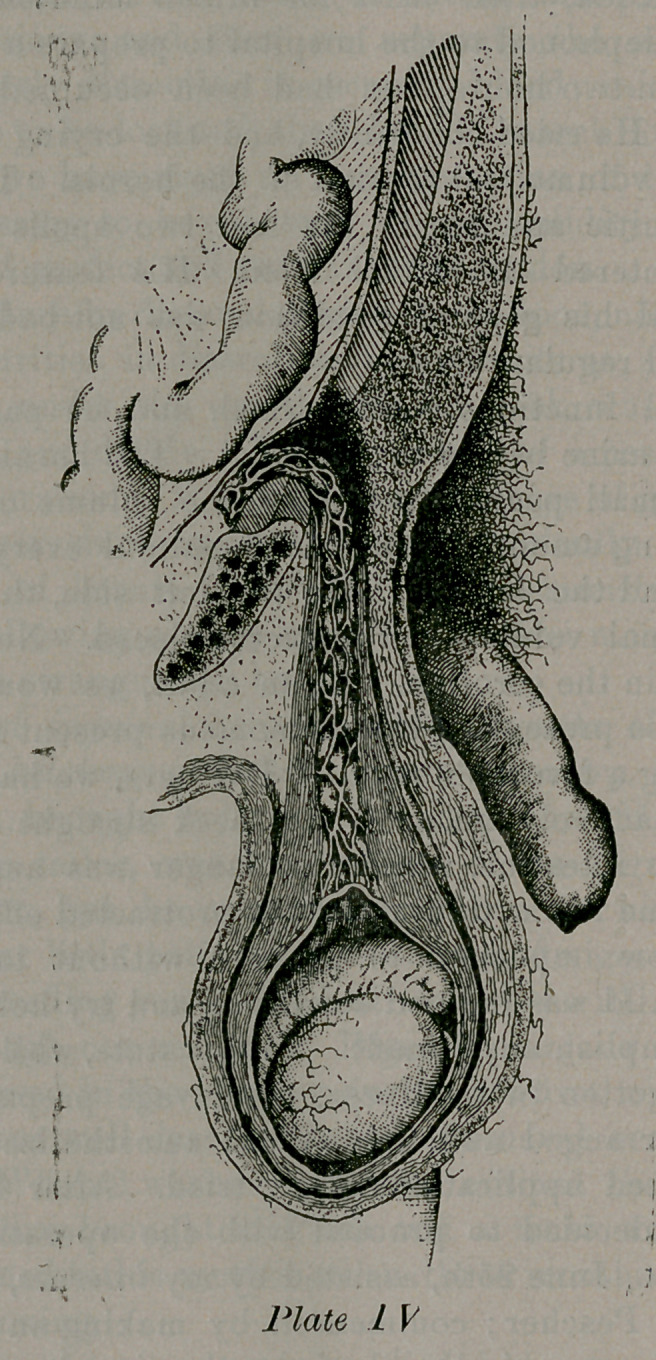# The Radical Cure of Hernia, Particularly with Children

**Published:** 1892-11

**Authors:** G. Felizet

**Affiliations:** Surgeon to Tenon Hospital, Paris, France


### THE RADICAL CURE OF HERNIA, PARTICULARLY WITH CHILDREN.

BY DR. G. FELIZET, SURGEON TO TENON HOSPITAL, PARIS, FRANCE.

Translated by THOS. H. MANLEY, A. M., M. D., Visiting Surgeon to Harlem Hospital, New York.

(*Continued.*)

We will often find the trouble attributable to the testis, when this organ has not descended into the scrotum. It is often possible, if the cord is supple and the testis has not contracted adhesions at the opening of the canal, to lower it and permit the intestine to pass. Sometimes force, gently applied, will bring the testis well down. It is here where we find suturing the ring useful. But the situation is sometimes very embarrassing, and many surgeons have not hesitated to castrate, under these circumstances. The intestine reduced, little remains to be done, but to complete the operation with ordinary precautions.

(*d*) The hernia is totally irreducible.

Herniæ totally irreducible by adhesions are not observed in children. It is in the adult, following repeated engorgements and pressure on hernial peritoneum, that we see established this unfortunate condition of things. The herniæ presented are not generally the "strangulated variety," but they are "accidental" herniæ, painful and troublesome, for which the operation for the radical cure is necessary, sooner or later.

For this order of cases, the employment of our apparel, the balloon, is useless and the dissection is long, difficult and perilous. It is because of the unequal and uncertain volume of the epiploon or intestine, the surgeon having no guide to the serous membrane of the sac. The enterprise is all the more difficult because of the thickening and change in consistence of this membrane by exudates and adhesions anteriorly.

Nothing is easier than to become bewildered in the incertitude of a dissection so obscure, or liable to bruise or wound the intestine. It is an affair requiring tact, patience, circumspection and the keen employment of sight, smell and touch. It requires a long time to complete the operation, many times an hour or more. But, as we have said, this type of adhesion is not encountered often in children, and is still less in infants; that is, in those who badly sustain surgical intervention of long duration.

THE RADICAL CURE AFTER KELOTOMY.

A patient is overtaken by the dangers of a strangulated hernia. Taxis not succeeding, we practice a kelotomy. The intestine is returned. The patient is cured; that is to say, his life is saved and he has survived his perilous experience. Sooner or later, the intestine again occupies the sac; the return of the hernia is almost inevitable, if we do nothing further than release the constriction.

The reproduction of the infirmity, eventually with all its former troubles, has in all times, pre-occupied the thoughts of herniotomists, and the list is long of the means imagined and tried, which would establish a definite cure after reduction of the protrusion.

We find in the classic treatise of En. Paul Legoud, a description most complete of many of the methods and apparatuses which have been conceived, invented and tried, to assure the radical cure of a hernial infirmity. We see, that in early times, the number of preventatives equal nearly the the number of appliances, yet at that time, the mortality was very high. Before the days of antiseptics in surgery, the length of time consumed in operation, the uncertainty of its results, the frequency of suppuration, the propagation of inflammation towards the peritoneum, the danger of killing the patient by operative manœuvre itself, the efficaciousness of which, at best, was more or less problematical; all seemed, during the past century and a half, or longer, to have rendered surgeons circumspect; so that even to-day, the masters who undertake the radical cure after kelotomy, are not infrequently ridiculed.

The actual tendencies at present, are greatly changed. Surgery of herniæs has become practically harmless in its boldness and our manœuvres; for radical cure do not add to the difficulties, the dangers which an elder generation of operators feared.

The operation for radical cure is not in truth, however, to be regarded as an audacious and reckless interference, or an enterprise of rashness and insurmountable difficulties.

It is wrong to pretend to perform a great operation for a condition which is not beyond the reach of every surgeon to succeed with. We may rest assured, as we have said, that it is an operation simple of execution; as mediocre qualities only are called for. Patience and good judgment are common pre-requisites, which represent, we might say, the authography of surgery. When we attentively observe the progress of those radical cures properly conducted, we are astonished with the advantageous statistics. With all eventualities anticipated, and ample care exercised, the death-rate will become almost *nil.* The difficult question is not, to not kill your patient, but to cure him of this infirmity. Now, we may completely cure a hernia when we deal properly with the neck and sac, but the result is not realized unless operated as high as possible.

In the operation, in the beginning, for radical cure, made the occasion of a kelotomy, the ablation of the sac is laborious, and it is to one's advantage to free the constriction, when we may easily extirpate an anatomically normal pedicle; but when we are in the presence of a membrane, red and inflamed by recent injury in attempts at reduction, adherent at the sides and underneath, and where the dissection is attended with free hemorrhage, we have a difficult, complicated case. There is considerable blood spilt and much time lost—the points on which we will later dwell—because they constitute two factors most principal in the way of success of operation in early infancy. We may add that the integrity of the neck is always altered after kelotomy; may be it has formed an unique bridle. One may sometimes in dealing with it, follow the counsel of Louis and Vidal (de Cassis), and practice small, radiated sections; and then we separate the serous membrane, tediously dissecting in escaping blood; raise the parts in fragments to be lost and then found; search for the neck, so that now we have more or less laceration of the sac, that part so necessary to have intact, to give solidity and guarantee of cure of perfect and permanent occlusion.

The procedure by distension with the balloon, not only clears up the obscurities in dissection, but permits us, also, to have immediately under the eye the sac and neck, which we ligate with cat-gut, and divide. It also permits of a neat, rapid and bloodless operation. It possesses advantages of greater value than any other since operations were originally devised for radical cure of hernia in children. To demonstrate this, we report the following case in an infant twelve months old, afflicted with a strangulated hernia.

*Stangulated inguinal hernia; twelve months old; repeated taxis failed; kelotomy and radical cure—recovery.*

Charles N., brought to Tenon Hospital, June 25, 1890. The father was a tailor, was the subject of an inguinal hernia since infancy; but he was the only one in his family so afflicted. Our little patient was healthy and vigorous, born at term the preceding year. He had never been ill. Never had whooping-cough nor measles. Digestive organs active. He had been nursed in the country during his first nine months. His testes were well descended, and his naval scar was well cicatrized. When 6 weeks old, it was noticed that he had a hernia. He was brought to Paris and a truss was adjusted; he was returned to his mother when three months old, his infirmity continuing.

Many different trusses were applied, but none could completely control the hernia. They all caused incessant pain, and it came down with great facility. The mother said when the hernia was returned, a distinct gurgling sound was audible. On June 24th, the hernia came down in great volume, and was much more indurated than usual. He cried constantly. The mother tried vainly to return it. A physician was called in, and resorted to various expedients; but they all failed. The Wednesday following, at 6 P. M., an *interne* at the Hotel Dieu, tried taxis for fifteen minutes, but failed. We then telephoned to the hospital to prepare a bed for him.

More than two hours then had been occupied with tentative taxis. He cried constantly, and the crying visibly augmented the vclume and tension of the hernia. The abdomen was tympanitic and hard. He had two spells of vomiting after he entered the Hotel Dieu. His features were not pinched and his general condition was not bad. The pulse was full and regular.

The renal functions were normal, and he passed a large quantity of urine before we operated. The hernia was of the size of a small pear (three times the volume of an infant's thumb), being smooth, regular and painful everywhere. We could not find the testicle. On the left side, the testis was felt of normal volume and correctly placed. No ecchymosis nor œdema in the scrotum. In an adult, we would be justified, in a case presenting the phenomena present in this case, of predicting a favorable result. But here, we had a congenital hernia; actively inflamed; its neck straight and tightly gripping the intestine, while the danger was augmented by length of time it was down, and the protracted efforts at taxis.

It was now important to proceed without loss of time, while the child was in good condition, and try kelotomy if we would accomplish any benefit. In this state, while everything was being gotten in readiness, the lavage prepared, the instruments arranged and the patient anæsthetized, moderate taxis and iced applications were tried. After five minutes trial, it was decided to proceed with the operation. Operation at 6 P. M., June 25th, assisted by my *internes*, M. M. Camascesse and Pescher; commenced by making an incision five or six centimetres in length, from the ring downward. But one ligature was necessary to secure an arteriole in the external lip of the incision.

We proceeded rapidly, and with two or three cuts of the scalpel, the sac was nearly exposed. It was seized and

*Plate IV*

opened with every precaution, and there escaped a half teaspoonful of coffee-colored serum. It was nearly a dry hernia. The opening was now enlarged with the scissors, and maintained with two forceps. We now saw that the serosa of the intestine had been nicked by the scissors, but it was so small it did not prevent direct reduction of the intestine. The finger was now introduced and carried to the base of the sac, where the testicle was recognized in its normal position. Taxis direct now applied, made no impression on the hernia; hence, Cooper's hernia bistoury was introduced before and posteriorly, and a section made of a firm hard body at the neck of the sac, well accentuated. Reduction was now made without difficulty; careful lavage of the sac and testis.

The intestine returned, the internal surface of the sac was wrinkled in longitudinal folds, velvety, and of a rosy hue; in fact, it bore a striking resemblance to the empty bladder. It is certain, that under ordinary management, the peritoneo-vaginal pedicle would give rise to free hemorrhage or division, while with the balloon, none was lost. The balloon being engaged in place of the withdrawn finger, its small extremity is carried up as far as possible in the inguinal tract; the wall closed, we insufflate gradually, so that a genuine ovoid tumor is formed. Now the separation of the elements of the cord is easy before we allow the artificial pouch to collapse. The separation of the neck of the sac was effected as high as possible, in such a manner as to avoid injury by the bistoury. Moderate tortion was then made followed by traction in a downward direction. It was ligatured by cat-gut. Section was made one centimetre below; the ligature disappeared instantly into the abdomen. The sac was reversed and excised. Sufficient of it was preserved below to vaginalize the testis. The hernial sac was homogeneous, thin and transparent.

It had the capacity of an egg and weighed twenty-eight centigrammes. Irrigation with boracic acid solution; suture with catgut; drain. Dry dressing with powdered salol; kelotomy, reconstitution of the sac; technique by radical cure, suture; all accomplished within twenty-five minutes.

Ten grammes of chloroform used. He lost ten grammes of blood. The subsequent dressings were exceedingly simple.

June 25: During the day no pain, no vomiting. Abdomen still hard, urinates freely. He is playful, drinks grog and milk, rectal temperature 38 degs. C., no gas nor evacuation.

June 27: Emits gas this morning. Abundant motion. We removed the dressings, fearing that they might be soiled by urine or fæces. There was nothing. The sutures hold well. The region is red and tumified; no pain; to be returned. Dry dressing with salol.

June 28: General state excellent; motion normal; temperature 38 degs. Truss removed. The youngster sitting up. Nothing more to note after this.

July 10: Left the hospital.

We last saw him July 29, when there was no more tumefaction, pain or re-appearance of a hernia.

### RESULTS CONSIDERABLE TIME AFTER THE OPERATION FOR THE RADICAL CURE OF HERNIA.

We have for a long time, a procedure which makes the operation for the radical cure of hernia, when practiced on simple or strangulated, an operation simple, easy and inoffensive, and we have explained a method which has a surgical advantage in giving precision and rapidity; besides economical in the loss of blood, which does not generally obtain, and which assures us the dissection of the peritoneal investment without extensive mutilation of the fibro-muscular tissue. It guarantees occlusion of the canal, strengthening of the track and suppression of the feeble point.

With minute precaution, we wish to be permitted to hope to realize a radical cure and definite result; "a hernia which exists no more and cannot return." Will we be disappointed in our hopes? We had twenty cases without a death, of which sixteen returned after a variable time, from fourteen months to three years. We have had two re-appearances complete in the scrotum; two re-appearances of hernia, not passing the groin; four incipient returns, and eight definite cures.

The eight relapses were of different degrees, with the sixteen cases which returned for examination. This figure, 50-100, represents the exact facts. It would scarcely express the full truth, however, because in sixteen cases the anatomico-pathological conditions were very dissimilar, as our clinical observation of them differed, and they had multiple features, although in their gross character were similar.

Age dominates prognosis, when the question of radical cure is considered. The results are more certain with the young adult than the old man, for with the latter the hernial track is capable of but little modification. Possibly the gap may fill in by an adventurous formation sufficient to sustain the pressure of the viscera, by the aid of the bandage, when a patient's condition is such as demands something to be done, as a temporary or tentative measure. The ring may be narrowed, which will all the more accentuate relief for a condition destined to be aggravated progressively in time. In those latter cases we may hope for much benefit from operation, as we can promise nothing.

It is otherwise in childhood. At this age, the young tissues, stretched by the passage of the intestines, offer us all the advantages of their suppleness. The pressure which distends them, at one time, often ultimately tends to efficiently restore the former normal position. The fibrous tissue in youth is wanting in that definate rigidity, neither that which is specially vitalized, nor the fasciæ or aponeurosis.

Finally, the irritation provoked in the operative region is active and rich in proliferation. This is not all; as one must, in appreciating the results of hernial operation during childhood and early life, take into account the absolute fact of the *natura naturans* of reparative vitality, he must also look to relative changes, occurring with age in this region proportionally as age advances.

Inguinal hernia is located in a triangle which has for its sides the arcade of Fallopius below; a part of the inferior extremity of the linea-alba, internally; externally, a horizontal line extending from the center of superior spine of the ilium to the linea-alba.

The longitudinal course of this triangle, in an adult of ordinary height, for example, being 100 centimeters and the face of the orifice being five centimeters long, one would say that the relation of the hernial opening at this part of the abdominal wall, is from 2, 5 p. 100. We can easily perceive, then, that in an adult operated on, the track being free from the assaults of a pressing hernia, the external orifice retracting the least, the relations would then be more than or thereabouts, 2, 5 p. 100.

With the child, and I do not wish to speak of the new-born, but with a child of six years, for example, I suppose that the face of the fictitious triangle would be thirty centimeters wide; the relation between the hernial opening and this part of the abdominal wall, would be more than with an adult, say from 6, 5 p. 100.

If the hernia, having been operated on and its orifice only retracting, the ratio after cure would be 3, 25 p. 100; but it must be admitted that the space in the triangle would not increase in the physiological development of the young subject. Now, we know that between the sixteenth and eighteenth year, the limits of the triangle more than double; and if we admit (and we are rather under estimating) that the external orifice may be diminished, as with the adult, we would have an opening one centimeter wide, on a triangle of seventy centimeters in its greatest length; that is to say, a relation from 1, 4 p. 100, instead of 3, 25 p. 100 with the surface of the triangle as we have indicated.

The child grows, and each year progressively diminishes, proportionally, the track, until its conformation and dimensions are normal. This is not all; for while the rent in the triangle is weak, it retracts by its relation to the surface of the abdominal wall the calibre of the intestine at its side in such a manner that one part of the track is most straight, and the other viscera having augmented in volume, the conditions which produce the hernia have become obliterated.

The result of a successful operation constitutes an obstacle. The natural development has been transformed, rendering a relapse an impossibility. To effect this impossibility, is the ideal objective in the radical cure of hernia, and indeed, it often does become a reality; thanks to the organization of a perfect obstacle, to the tissue of the intestine. We have long explained this as the perfection of operation, which leads to the permanent effacement of the peritoneal funnel and the constitution of a solid support. Now, it is by the insufficiency of this support that we have relapses.

*(To be continued.)*

## Figures and Tables

**Plate IV. f1:**